# Evaluation of Development of Nonalcoholic Fatty Pancreas Disease After Post-Endoscopic Retrograde Cholangiopancreatography Pancreatitis in Liver Transplant Patients: Computerized Tomography Versus Ultrasound

**DOI:** 10.5152/tjg.2023.22424

**Published:** 2023-11-01

**Authors:** Ayşe Nuransoy Cengiz, Yılmaz Bilgiç, Sinan Karatoprak, Ayşe Gökçe, Bahri Evren, Sami Akbulut, Sezai Yılmaz

**Affiliations:** 1 Division of Internal Medicine, Kahramanmaraş Elbistan State Hospital, Kahramanmaraş, Turkey; 2 Division of Internal Medicine, Department of Gastroenterology, İnönü University Faculty of Medicine, Turgut Özal Medical Center, Malatya, Turkey; 3 Department of Radiology, İnönü University Faculty of Medicine, Turgut Özal Medical Center, Malatya, Turkey; 4 Department of Public Health, İnönü University Faculty of Medicine, Turgut Özal Medical Center, Malatya, Turkey; 5 Department of Endocrinology, İnönü University Faculty of Medicine, Turgut Özal Medical Center, Malatya, Turkey; 6 Department of Surgery and Liver Transplant Institute, Turgut Özal Medical Center, Malatya, Turkey

**Keywords:** Ultrasound, liver transplantation, PEP, NAFLD, NAFPD

## Abstract

**Background/Aims:**

Post-endoscopic retrograde cholangiopancreatography pancreatitis (PEP) is the most common serious adverse event in liver transplant patients The average incidence has been reported as 1.3%-15.1% in prospective series. In our study, we have prospectively evaluated the prevalence of nonalcoholic fatty pancreatic disease (NAFPD) after PEP via computerized tomography (CT) and determined the ratio of fatty pancreas by ultrasound imaging in liver transplant patients.

**Materials and Methods:**

We have retrospectively analyzed 2922 patient files, and 146 patients were indicated for ERCP. PEP was observed in 32 patients. After presenting the significant association between the NAFPD and PEP, we prospectively reached 32 patients included in the study. Ten out of those patients have been performed ultrasound with regard to NAFPD.

**Results:**

PEP was defined in 32 patients in whom CT was performed to investigate NAFPD. When the patients were contacted, it was observed that 12% were deceased, 71% were alive, but 15% of them were untraceable. Ultrasound has been performed on 10 of 32 patients to determine NAFPD. There was a significant reduction in post-PEP pancreas/spleen rate compared to pre-PEP pancreas/spleen rate (*P *= .001). Both the pre-PEP and post-PEP pancreas-spleen difference dropped significantly (*P *= .002).

**Conclusion:**

Ultrasound imaging could be utilized as a scanning test and an alternative to evaluate and diagnose NAFPD, particularly in risky patients.

**Main Points**

- In our study, we evaluated the prevalence of nonalcoholic fatty pancreatic disease (NAFPD) after post-endoscopic retrograde cholangiopancreatography pancreatitis (PEP) in liver transplant patients.
- We determined prospectively how many of the patients in this group also saw fatty pancreas by ultrasound (US).
- The NAFPD can be considered in the post-PEP course of patients, and such simple, practical, and affordable diagnosis and scanning test as US count be used to track those patients.

## Introduction

Fatty pancreas was first described in 1933^1^, as prominently one of the topical subjects of gastroenterology. Previous research on this subject is very limited, and as of March 2022, only 313 studies have been identified on PubMed. Several diseases such as acute pancreatitis, pancreatic carcinoma, and chronic pancreatitis cause the death of acinar cells which trigger inflammation and as a result nonalcoholic fatty pancreatic disease (NAFPD) development can be observed.^2,3^

Post-endoscopic retrograde cholangiopancreatography pancreatitis (PEP) is the most common serious adverse event in liver transplant patients.^4,5^ Although the mechanisms are not clear, PEP is considered to occur due to an inflammatory cascade triggered by injury to the acinar cells that leads to the systemic release of cytokines.^6^ Among these, pancreatic fat is gaining attention as the likely risk factor for acute pancreatitis.^7^

There are some studies on the severity of NAFLD and pancreatitis.^8,9^ In addition, there are few studies on NAFLD claiming that it was the strongest predictor of PEP.^9,10^ However, no research has ever been conductedon the occurrence of fatty pancreas after PEP.

In this study, we tried to elucidate the prevalence of NAFPD after PEP in liver transplant patients by computerized tomography (CT) and determine the ratio of fatty pancreas by ultrasound (USG) imaging in liver transplant patients.

## Materials and Methods

### Subjects and Study Design

The reviewed data is from the registration database of the Liver Transplantation Institute and Gastroenterology units of Turgut Özal Medical Center, which is a tertiary health institution. In total, 2922 living donor liver transplantations (LDLT) were performed in our center between March 2002 and March 2021.

A total of 2922 patient files were evaluated retrospectively in accordance with the Helsinki Declaration decisions, patient rights regulations, and ethical rules based on the approval from the İnönü University Clinical Research Ethics Committee (Approval number: 2021/2057). The following inclusion criteria were used for all reviewed medical records included in the study:

(i) Liver transplantation patients 18 years old or over with CT performed at Turgut Özal Medical Center between 01 March 2002 and 01 March 2021 were included, and (ii) the patients were followed up for at least 6 months.

**Figure 1.**
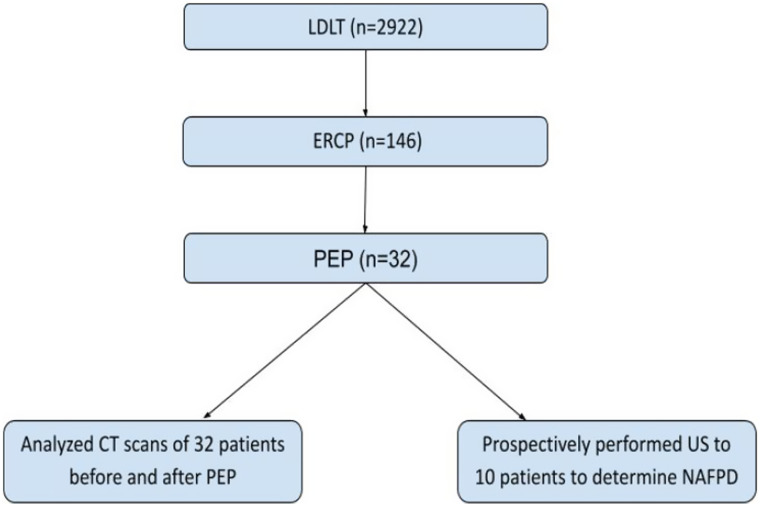
The study population obtained in the registration database.

We have retrospectively analyzed patients and recognized that 146 patients were indicated for ERCP due to various reasons. Post-endoscopic retrograde cholangiopancreatography pancreatitis was observed in 32 patients. The PEP was defined as the onset of abdominal pain more than 24 hours after the procedure, lasting more than 4 hours, with high serum levels of amylase at least 3 times the upper limit of normal and patient admission was required.^11^ We retrospectively analyzed these 32 patients’ CT scans performed after PEP to investigate NAFPD. Having reached these 32 patients, we found that 4 of them had deceased and 18 of them were followed up in different centers. Hence, we have prospectively performed USG on 10 patients to determine NAFPD (Figure 1).

All medical records of eligible patients were retrospectively reviewed and the following parameters were collected: demographic data (age, gender, transplantation etiology, previous endoscopic retrograde cholangiopancreato­graphy (ERCP), antibiotic use), clinical presentation [cause of ERCP, previous endoscopic sphincterotomy (EST)], endoscopic details (papillotomy, balloon dilation, stent insertion, canalization of pancreatic duct), as well as imaging information regarding the liver per abdominal USG and/or abdominal CT were also collected from eligible patients records.

### Statistical Analysis

Statistical Package for the Social Sciences vs. 22.0 (IBM Corp.; Armonk, NY, USA) has been utilized for statistical analyses. The data were expressed as n (number) and % (percent). While considering the data in the study, median (min-max) was used in the variables. Shapiro–Wilk test of conformity to normal distribution was used. It was observed that the variable of pancreas/spleen (P/S) rate before PEP was not compliant with normal distribution (*P *< .05). PS difference before PEP, P/S ratio after PEP, and P-S difference after PEP were found to be compliant with the normal distribution (*P *> .05). Wilcoxon test was performed for statistical analyses while the significance level was accepted as *P *< .05.

### Radiological Analysis

The CT images were acquired using the 256-section multi-detector tomography equipment (SOMATOM Definition Flash, Siemens Healthineers, Forchheim, Germany). The attenuation values in the pancreatic tissue were calculated in terms of Hounsfield Unit, using a 1 cm^2^ region of interest area. In an effort to standardize the attenuation values in the pancreatic tissue, the attenuation of the spleen tissue was measured alike and pancreas/spleen (P/S) ratio and pancreas-spleen (P-S) difference was used. While specifying the points of measurement, the preferred sites were distal to the pancreatic channel, peripheral tissue, or vascular structures. The patients having a P/S ratio of lower than 70% were assumed to be pancreatic lipoidosis.^12^ Prospective applications of the patients with pancreatic lipoidosis were asked while the lipoidosis was evaluated in the pancreatic tissue using GE Logic S8 US machine (GE Medical Systems) with a 4 mHz curvilinear probe.

## Results

### Baseline Characteristics of Study Patients

A total of 32 patients were diagnosed with PEP, of which 59% were male and 13% were female. The average age of the patients was 56 years old. Thirty-two patients were retrospectively analyzed, and CT scans were performed after PEP to observe NAFPD. When the patients were contacted, it was observed that 12% were deceased, 71% were alive, but 15% of them were untraceable. Ultrasound has been performed on 10 of 32 patients to determine NAFPD (Table 1).

According to liver transplantation etiologies, hepatitis B virus was the most common cause and was almost observed in half of the patients (53%), followed by cryptogenic hepatitis (15%), fulminant hepatitis (12%), Wilson disease (9%), and hepatitis C virus with 3% (Table 1). Liver transplant anastomosis type, previous EST, canalization of pancreatic duct, antibiotics usage, ERCP indication, and stent status of patients are elaborated in Table 1.

**Figure 2:**
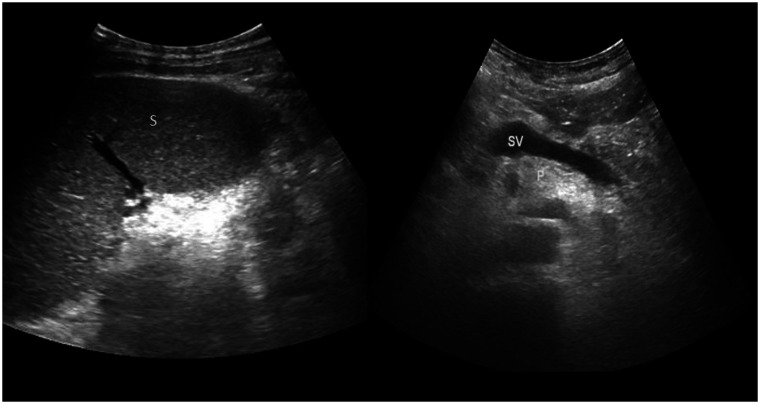
60 years old male who underwent LDLT due to fulminant liver failure and was performed ERCP due to biliary stenosis. Ultrasound image shows increased echogenicity of pancreas parenchyma compared with spleen. P, pancreas; S, spleen; SV, splenic vein.

The USG has been performed in 10 out of 32 patients and 3% of those patients had grade 3 NAFPD, 18% had grade 2 NAFPD, and 9% had grade 1 NAFPD. No NAFPD was observed in 6% (Figure 2). In terms of NAFLD, 18% of the patients were grade 2, 6% were grade 1, and 12% were normal. Both pancreatic and liver lipoidosis were observed in 18.75% of the individuals and only 6.25% of them had NAFLD while 9.37% had NAFPD (Table 2). Considering the CT images of the 32 patients who were included in the study, in an effort to standardize the attenuation values in the PEP pancreatic tissue, the attenuation of the spleen tissue was measured likewise and pancreas/spleen (P/S) ratio and pancreas − spleen (P-S) difference was employed. During the determination of measurement points, the ones distal to the pancreatic duct, peripheral tissue and vascular structure were preferred. The patients with a P/S rate lower than 70% were assumed to have NAFPD. The pre-PEP P-S difference average was −4.00 ± 4.12, pre-PEP P/S rate average was 0.90 ± 0.89, post-PEP P-S difference average was −11.83 ± 11.46, and post-PEP P/S rate difference was 0.74 ± 0.24 (Table 3).

**Figure 3.**
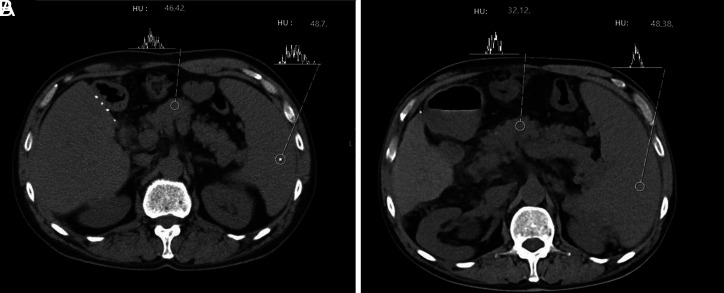
(A) Non-contrast CT image of the same patient in figure 2 shows attenuation values of pancreas (P) as 46 HU and spleen (S) as 48 HU. P-S difference: -2; P/S ratio: 0.96. (B) Control non-contrast CT after acute pancreatitis shows the same values as 32 HU and 48 HU respectively. P-S difference: -16; P/S ratio: 0.67. This ratio is compatible with fatty pancreas.

The differences in the post-PEP P/S rate and P-S difference were determined. The pre-PEP and the post-PEP pancreatic tissues were compared revealing a statistically significant change in the pancreatic tissue in favor of lipoidosis (Figure 3). There was a significant reduction in the post-PEP P/S rate compared to the pre-PEP P/S rate (*P* = .001). Both the pre-PEP and post-PEP P-S differences dropped significantly (*P* = .002), (Table 4).

The USG and CT radiological methods were compared for the diagnosis of NAFPD. The USG imaging was considered a new method while CT was accepted as a reference. Ten patients who received both USG and CT were included in the comparison for the purposes of calculating the sensitivity and specificity of the imaging techniques. The sensitivity of USG in detecting NAFPD was determined as 100%. In other words, all patients diagnosed by CT were also confirmed through USG. However, the specificity of USG was 28.5%, in other words low. The USG would therefore be a good scanning test for the diagnosis of NAFPD (Table 5).

## Discussion

Turkey is now the leading European country in LDLT with more than 1500 cases during the last 15 years.^13^ Our institution is extremely experienced in liver transplantation and its complications. Pancreatitis remains the most frequent complication of ERCP.^4^ Although the mechanisms are not clear, PEP is considered to occur due to an inflammatory cascade triggered by injury to the acinar cells that leads to the systemic release of cytokines.^6^ Among these, pancreatic fat is gaining attention as the likely risk factor for acute pancreatitis^7^ addressed by numerous studies on this topic. There were several studies on the severity of NAFLD and pancreatitis^8,9^ and in addition, a few studies claimed that PEP was the strongest predictor of NAFLD.^10^ On the other hand, there was no published study in the literature about the occurrence of fatty pancreas after PEP. We have retrospectively analyzed 2922 LDLT patients and 146 patients were indicated for ERCP for various reasons (Table 1). The PEP was observed in 32 patients.

The NAFPD was compared in the pre-PEP and post-PEP images of the 32 patients included in the study. With regard to the CT images taken during the post-PEP follow-ups, pancreatic steatosis was observed which was statistically significant compared with the former ones. The reason for this could be acinar cell loss in pancreatic tissue through inflammation.^2^ Also, 75% of our patients were indicated for ERCP because of the stenosis while almost 72% received stent within the procedure. This could be elaborated as a high-risk patient population due to the fact that our target population was transplant patients.

The first conclusion we have derived from this study was the increased frequency of NAFPD development in transplant patients. Although research on this subject is still ongoing, there was no study in the literature with regard to post-PEP NAFPD development. This conclusion is important with regard to clinical follow-ups. The patients could be considered also with regard to NAFPD in the post-PEP patient follow-ups. The NAFPD development in risky patients particularly in the obese group can be tracked, as NAFPD has been suggested to have a role in type 2 diabetes mellitus, acute pancreatitis, pancreatic cancer, and the formation of pancreatic fistula after pancreatic surgery.^7^

Moreover, unlike our hypothesis, there are studies on PEP development in patients with NAFPD in the literature. The yields however are different. Pokhrel et al stated that NAFPD was not a risk factor for PEP. In that article, Pokhrel et al identified 47 patients who developed PEP with a magnetic resonance imaging within 60 days of their ERCP, and in the end, they suggested that PEP-increased pancreatic fat content does not increase the risk of post-procedure pancreatitis.^12^ In another paper, Park et al defined NAFPD as a risk factor for the development of PEP.^9^ As a matter of fact, future studies with larger sample sizes are required to achieve statistically significant outcomes.

In our study, after presenting the significant association between the NAFPD and PEP, we prospectively reached 32 patients USG was performed on 10 out of 32 individuals with regard to NAFPD. It is known that USG failed to provide sufficient imaging, particularly in obese patients. Also, pancreatic fibrosis and NAFPD have similar appearances.^7,14^

To summarize, USG imaging does not provide sufficient evidence for NAFPD diagnosis, while its employment as a scanning test could be taken into account, as USG is easily accessible and affordable.^15^ In our study, the diagnosis rate of NAFPD via USG was found to be 100%. The USG can therefore be used as a scanning test. It can be an alternative to evaluate and diagnose NAFPD, particularly in risky patients.

Today, there are many studies on NAFLD in the literature. Also, there are studies as to its affecting the severity of acute pancreatitis.^8^ However no sufficient pathophysiology or clinical outcomes on NAFPD have been defined yet. We need application guides as to whether or not NAFPD is a benign pancreatic condition as well as diagnosis, tracking, or management. In the current study, we have investigated post-PEP NAFPD development status and the position of USG in scanning and diagnosis in difficult patient groups such as liver transplants. 

## Conclusion

As a result, our study is original and clinically supported with 2 perspectives: NAFPD can be considered in the post-PEP course of patients and USG imaging could be utilized as a scanning test and an alternative to evaluate and diagnose NAFPD, particularly in risky patients.

## Figures and Tables

**Table 1.** Distribution of Baseline Variables of the Patients

Age (minimum-median-maximum)	21-56-69	
	n	%
Gender		
Male	19	59.4
Female	13	40.6
Patient’s final status		
Exitus	4	12.5
Alive	23	71.9
Unknown	5	15.6
Anastomosis type		
Normal	6	18.8
Stenosis	24	75.0
Leakage	1	3.1
Stone	1	3.1
Previous EST		
None	13	40.6
Yes	9	28.1
Done in session with PEP	10	31.3
Canalization of pancreatic duct		
None	20	62.5
Yes	12	37.5
Transplantation etiology		
Hepatitis B virus	17	53.1
Hepatitis C virus	1	3.1
Toxic hepatitis	1	3.1
Cryptogenic hepatitis	5	15.6
Fulminant hepatitis	4	12.5
Wilson	3	9.4
Crigler–Najjar	1	3.1
Antibiotics usage		
None	14	43.8
Yes	18	56.3
ERCP indication		
Control	1	3.1
Stenosis	24	75.0
Leakage	3	9.4
Stricture	2	6.3
Stone	1	3.1
Stent status		
None	9	28.1
Yes	23	71.9

ERCP, endoscopic retrograde cholangiopancreatography; EST, endoscopic sphincterotomy; PEP, post-endoscopic retrograde cholangiopancreatography pancreatitis.

**Table 2.** Status of Liver and Pancreas USG Findings

	n	%
Pancreas US		
Normal	2	6.3
Grade 1	3	9.4
Grade 2	6	18.8
Grade 3	1	3.1
Unknown	16	50
Exitus	4	12.5
Liver US		
Normal	4	12.5
Grade 1	2	6.3
Grade 2	6	18.8
Unknown	16	50.0
Exitus	4	12.5

USG, ultrasound.

**Table 3.** Minimum, Median, and Maximum Values of Pre- and Post-PEP Variables

	Minimum	Median	Maximum
Pre-PEP difference	−10.0	−5.0	10.0
Pre-PEP P/S ratio	0.71	0.90	1.20
Post-PEP P-S difference	−45.0	−10.0	1.0
Post-PEP P/S ratio	0.03	0.78	1.00
	Average ± SS		
Pre-PEP P-S difference	−4.00 ± 4.12		
Pre-PEP P/S ratio	0.90 ± 0.89		
Post-PEP P-S difference	−11.83 ± 11.46		
Post-PEP P/S ratio	0.74 ± 0.24		

Post-PEP, post-post-endoscopic retrograde cholangiopancreatography pancreatitis; Pre-PEP, pre-post-endoscopic retrograde cholangiopancreatography pancreatitis; P-S difference, pancreas-spleen difference; P/S ratio: pancreas/spleen ratio.

**Table 5.** The Sensitivity and Specificity of USG Imaging

		CT		
		Lipoidosis Exists	No Lipoidosis	Total
US	NAFPD exists	3	5	8
	No NAFPD	0	2	2
	Total	3	7	10

CT, computerized tomography; NAFPD, nonalcoholic fatty pancreas disease; US: ultrasound.

Sensitivity: 3/3 × 100 = 100% US’s rate of diagnosis in comparison to CT.

Specificity: 2/7 × 100 = 28.5% US’s finding rate of the healthiest according to CT.

**Table 4.** Pre- and Post-AP P/S Rate Comparison of the Patients

Pre- and post-PEP P/S rate
			*P* ^*^
Negative Rank	19	.001
	Positive Rank	
	5	
Equal
0
Pre- and post-PEP P-S difference
	Negative Rank	16	.002
		Positive Rank	
8			
Equal
0

Pre-PEP, pre-post-endoscopic retrograde cholangiopancreatography pancreatitis; Post-PEP, post-post-endoscopic retrograde cholangiopancreato­graphy pancreatitis.

^*^Wilcoxon Marked Ranks Test.
